# A VCSEL-Based NIR Transillumination System for Morpho-Functional Imaging

**DOI:** 10.3390/s19040851

**Published:** 2019-02-19

**Authors:** Sabina Merlo, Valentina Bello, Elisabetta Bodo, Sara Pizzurro

**Affiliations:** Dipartimento di Ingegneria Industriale e dell’Informazione, Università di Pavia, 27100 Pavia, Italy; valentina.bello01@universitadipavia.it (V.B.); elisabetta.bodo01@universitadipavia.it (E.B.); sara.pizzurro01@universitadipavia.it (S.P.)

**Keywords:** transillumination, VCSEL, near infrared radiation, non-ionizing radiation, tissue imaging, non-invasive morpho-functional imaging

## Abstract

Transillumination with non-ionizing radiation followed by the observation of transmitted and diffused light is the simplest, and probably the oldest method to obtain qualitative information on the internal structure of tissues or body sections. Although scattering precludes formation of high-definition image (unless complex techniques are employed), low resolution pictures complemented by information on the functional condition of the living sample can be extracted. In this context, we have investigated a portable optoelectronic instrumental configuration for efficient transillumination and image detection, even in ambient day-light, of in vivo samples with thickness up to 5 cm, sufficient for visualizing macroscopic structures. Tissue illumination is obtained with an extended source consisting in a matrix of 36 near infrared Vertical Cavity Surface Emitting Lasers (VCSELs) that is powered by a custom designed low-voltage current driver. In addition to the successful acquisition of morphological images of the hand dorsal vein pattern, functional detection of physiological parameters (breath and hearth rate) is achieved non-invasively by means of a monochrome camera, with a Complementary Metal Oxide Semiconductor (CMOS) sensor, turned into a wavelength selective image detector using narrow-band optical filtering.

## 1. Introduction

Optical imaging methods have been successfully applied in biomedical and clinical applications and reviewed in several recent publications [1,2,3]. Among them, optical transillumination is a non-invasive diagnostic technique based on the use of a light source placed underneath the tissue under test, to identify internal structural abnormalities. After crossing the tissue, light appears to the observer more or less intense, depending on the attenuation due to absorption and scattering. 

Whereas transillumination is widely used in optical microscopy of thin samples, light absorption and scattering of tissues impose severe limitations on the penetration depth when dealing with thick specimens. If light is absorbed and not reemitted, photons are lost and a dark image is recorded. In biological tissues, the absorption of the radiation is mainly caused by water, present in large amount, and by macromolecules, such as proteins, and pigments, such as melanin and hemoglobin [4,5]. While local absorption properties govern light-tissue interactions, average absorption properties govern light transport. In the red and near infrared wavelength range, that extends from 600 to 1200 nm, water absorption is minimal: this region is often indicated as “diagnostic and therapeutic window” and radiation can penetrate in more depth, if compared to the visible [6]. It is still strongly absorbed locally by hemoglobin in vessels. With regard to scattering, low level light is merely redirected but can still be detected. This effect precludes formation of high definition images, unless complex tomographic and gating techniques are employed [7,8,9], that are anyway suitable only for analyzing limited surface area. Transillumination based on the detection of scattered photons that are able to reach the detector after propagation through the tissue still allows to reconstruct images with details on the inner structure of wide tissue areas but with lower resolution than that achieved with techniques based on ionizing radiations.

For all the reasons mentioned so far, transillumination followed by the observation of diffused light, though probably the oldest and simplest method to obtain qualitative information on the internal structure of tissues or of a body section [10,11,12,13], is not yet considered a reliable diagnostic method. Thus, it is only exploited as a first approach to a diagnostic suspicion, in view of more detailed and invasive tests [14,15]. Transillumination for diaphanoscopy is applied in the biomedical field for identifying hydrocele, hydrocephalus, caries or other dental damages, malignant lumps as well as vein locations [16]. Medical devices based on transillumination that are commercially available, such as Astodia and Breastlight, employ visible light radiation, mainly red, and can be used only in a darkened place [17,18]. They are based on the use of single light emitting devices, such as LEDs, eventually at more than just one wavelength. They do not envisage a capture option for image/video storage and, therefore, the outcome is based only on personal human perception. 

Transillumination is also functional to transmissive photoplethysmography (PPG), a non-invasive optical technique that provides a signal related to the changes in blood volume in the tissue under observation, typically finger or ear lobe [19,20]. Recent studies emphasize the potential information embedded in the PPG waveform signal and it deserves further attention for its possible applications beyond pulse oximetry. PPG is considered a promising technique for early screening of various pathologies but a full understanding of the diagnostic value of the different features is still lacking. Although the morphology of the fingertip PPG signal obtained with pulse oximeters looks similar to the arterial pressure pulse, the wave contour is not the same. The fingertip blood vessels contain alpha adrenergic receptors which affect the arteries and veins vasoconstriction (narrowing the blood vessels). Sensor pressure on the finger (or the ear lobe) might affect the blood flow and thus the shape of the recorded wave. Time-varying signals provided by a transillumination video system designed for image detection could be exploited for extraction of vital sign such as heart beat rate or respiratory rate or even pulse pressure wave [21]. Currently available systems based on transillumination are optimized either for imaging purposes of wide surface area or for detecting time dependent parameters, by illuminating a very narrow region on the sample. 

Amazing advancements have been done in the past years in the fabrication technology of compact, efficient, reliable optical sources with emission spectrum in the wavelength region 800–1000 nm as well as of image photodetectors highly sensitive in the same spectral range [22,23,24,25]. For example, Vertical Cavity Surface Emitting Lasers (VCSELs) are now fabricated with great features such as low threshold currents for high efficiency and low power consumption, surface-normal emission, low divergence and circular output beam, scalability to one- or two-dimensional arrays, very high speed, controlled single transversal mode and polarization, good wavelength stability. 

In this work, in view of the technological advances, we have reconsidered transillumination for morpho-functional imaging of in vivo tissues. We here describe the portable, optoelectronic instrumental configuration that we have assembled and tested to achieve efficient transillumination and image detection, even in ambient day-light, of morphological details of diffusing non-homogeneous biological samples with thickness up to 5 cm, with a resolution of the order of a few millimeters, sufficient for differentiating internal macroscopic tissue structures. Tissue illumination is obtained with an extended source consisting in a matrix of 36 near infrared VCSELs that is powered by a custom designed low-voltage current driver. Images of the vascular hand tree featuring good contrast are acquired non-invasively with a monochrome Complementary Metal Oxide Semiconductor (CMOS) camera turned into a wavelength selective image detector using optical filters with a 10-nm-wide transmission band. As a technical proof of principle, to demonstrate the potentiality of this system for generating low resolution images of in vivo human tissues, it has been employed for transilluminating human hands. The collected images, taken even in ambient day-light conditions, provide a clear visualization of the main blood vessel pattern. Detection of vital signs, exploiting data acquisition capability in the time domain, has been demonstrated by processing short video sequences that allowed heart and breath rate to be obtained without applying any physical constraint or thermal stress on the tissues.

## 2. Instrumental Configuration of the Infrared Transillumination System

**Figure 1:**
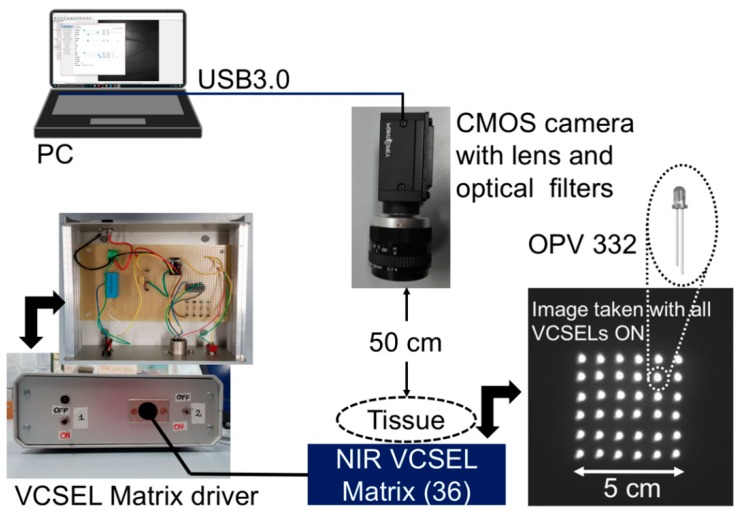
Instrumental configuration of the portable system for infrared transillumination.

The instrumental configuration of the transillumination system for functional imaging includes our custom-designed infrared illuminator and a digital monochrome CMOS camera for image acquisition, as well as a notebook for data acquisition and processing (Figure 1). The illuminator consists in a square matrix of 36 VCSELs (OPV 332, OPTEK Technology, TTElectronics, Woking, UK) that are firmly assembled in an aluminum plate with 36 mounting holes drilled side by side on a 5 cm by 5 cm area. The VCSELs exhibit multimode spectra characterized by a nominal peak emission wavelength at 850 nm and an optical bandwidth of 0.85 nm. This emission spectrum is located within the diagnostic and therapeutic window that is the range of wavelengths where light has its maximum depth of penetration in tissue due to the low water absorption and experiences substantial effects of local absorption in blood vessels due to hemoglobin. The dome lens of their T-1 packaging allows to generate a narrow beam with full-angle divergence of 4° [26]. The intrinsic directionality of the laser beam is impaired by light scattering induced by tissues when the laser radiation is traveling through the biological medium; the limb under test is transilluminated also by diffused light. Each VCSEL, driven at the operating DC current of 11 mA, provides an optical power of approximately 6 mW with a forward voltage of 2.2 V. By considering the power emitted by each VCSEL, the low divergence angle and the wavelength, we have verified that the emitted radiation is safe and should not induce any damage to the illuminated skin. 

**Figure 2:**
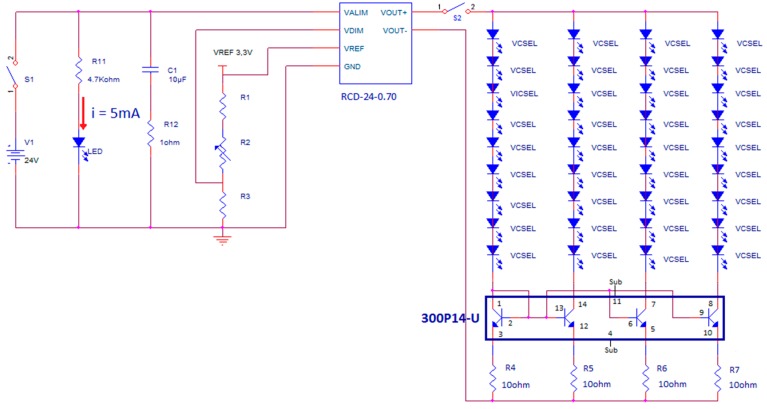
Schematic of the dedicated circuit for current driving the VCSEL matrix.

The VCSEL matrix is current driven by a dedicated circuit, placed in an Al case with input and output electrical connectors, that is powered by 24 V DC voltage obtained with a transformer from the main 220 V, 50 Hz line, to comply with safety requirements of medical devices. Figure 1 illustrates, on the left, a view of the front panel and of the content (electronic board) of the Al case of the current driver. Figure 1 also shows, on the right, a picture of the aluminum plate with all the VCSELs on, taken directly with the CMOS camera, without any object or obstacle in between. The circuit scheme is shown in Figure 2. A current mirror, with four branches, each one driving a series of nine VCSELs, is employed for biasing all the 36 VCSELs and it incorporates a matched-transistor monolithic array (THAT 300P14-U, THAT Corporation, Milford, MA, USA) with four matched NPN transistors. A current driver (RCD-24-0.70-Vref, RECOM, Gmunden, Austria) generates the required total DC current (11 mA by four, thus 44 mA total) by setting the resistance value of a trimmer (R2 in Figure 2).

**Figure 3:**
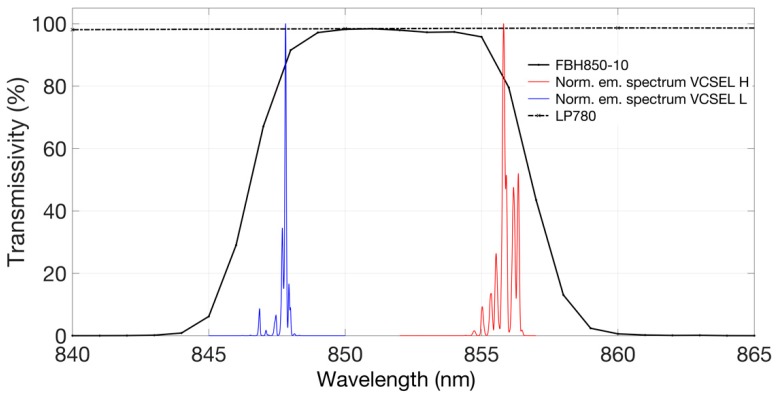
Comparison between the transmittivity spectrum of the optical filters (black traces) and the normalized emission spectra of two samples of the OPV 332 VCSELs, both driven at 11 mA. The red trace (VCSEL H) is the normalized emission spectrum of a VCSEL sample with the highest value of peak emission wavelength (among all the available VCSEL samples) whereas the blue trace refers to the normalized emission spectrum of the VCSEL sample with the lowest value of peak emission wavelength. Both spectra are contained within the transmission band of the narrow bandpass filter FBH-850-10. All the tested samples of OPV 332 VCSELs exhibited spectra with the emission peak falling inside the wavelength range from 847 up to 857 nm.

Acquisition of pictures and of compressed M-PEG videos is performed by means of a Near InfraRed (NIR) enhanced, monochrome CMOS camera (GS3-U3-41C6NIR-C, CMOS sensor 1”, 2048 × 2048 pixels, Point Grey Research Inc., Richmond, BC, Canada) equipped with an Electrophysics TV Lens (25 mm, 1:1.4). A long-wavelength-pass optical filter with 780 nm cut-on wavelength (MidOpt LP780, Midwest Optical Systems, Inc., Palatine, IL, USA) and a 10-nm-bandpass optical filter centered at 850 nm (FBH850-10, Thorlabs Inc., Newton, NJ, USA) are placed in front of the camera to selectively detect the diffused radiation at the wavelengths emitted by the illuminator and to efficiently reject visible and infrared ambient light. The camera is USB3.0-interfaced to a notebook using dedicated software (FlyCapture2, Point Grey Research Inc.). During the measurements, the volunteers were asked to place their limb upon the VCSEL matrix, underneath the camera, which was mounted on a tripod at a distance of approximately 50 cm from the skin surface. Figure 1, on the right, presents a picture of the VCSEL matrix, with all laser on, taken with the CMOS camera, which clearly shows the corresponding light spots. As the OPV 332 VCSELs are relatively low cost, standard commercial devices, the values of their effective peak emission wavelength at the operating current are distributed in an approximately 10 nm range. In Figure 3, we report the comparison of the transmittivity spectra of both optical filters (black traces) and the normalized emission spectrum of two samples of OPV 332 VCSELs, both driven at 11 mA.

The red trace (VCSEL H) represents the normalized emission spectrum of a VCSEL sample with the highest (H) value of peak emission wavelength (among all the available VCSEL samples) whereas the blue trace corresponds to the normalized emission spectrum of the VCSEL sample with the lowest (L) value of peak emission wavelength. Both spectra are contained within the transmission band of the narrow bandpass filter FBH-850-10 that allows very good rejection of ambient light and very low attenuation of the NIR photons. All the tested samples of OPV 332 VCSELs exhibited spectra with the emission peak falling inside the wavelength range from 847 up to 857 nm.

## 3. Results

**Figure 4:**
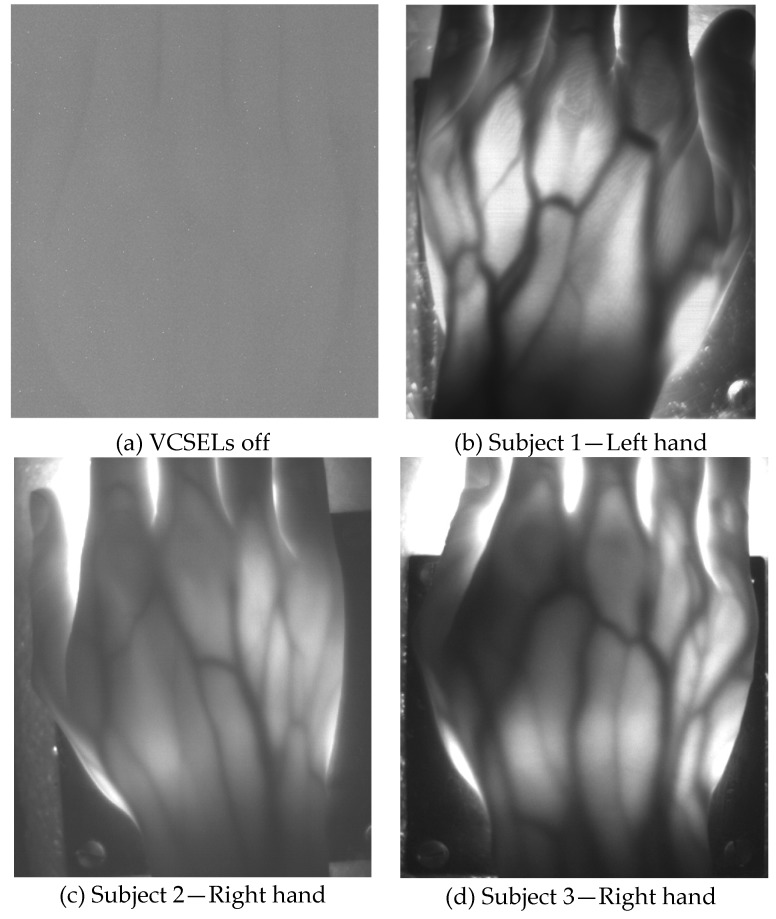
Raw pictures acquired with the transillumination system on white-skin, female subjects 1, 2 and 3, in standard ambient day-light. (**a**) Picture acquired with VCSELs off; (**b**)–(**d**) Pictures acquired with VCSELs on, where morphological details are well visible.

**Figure 5:**
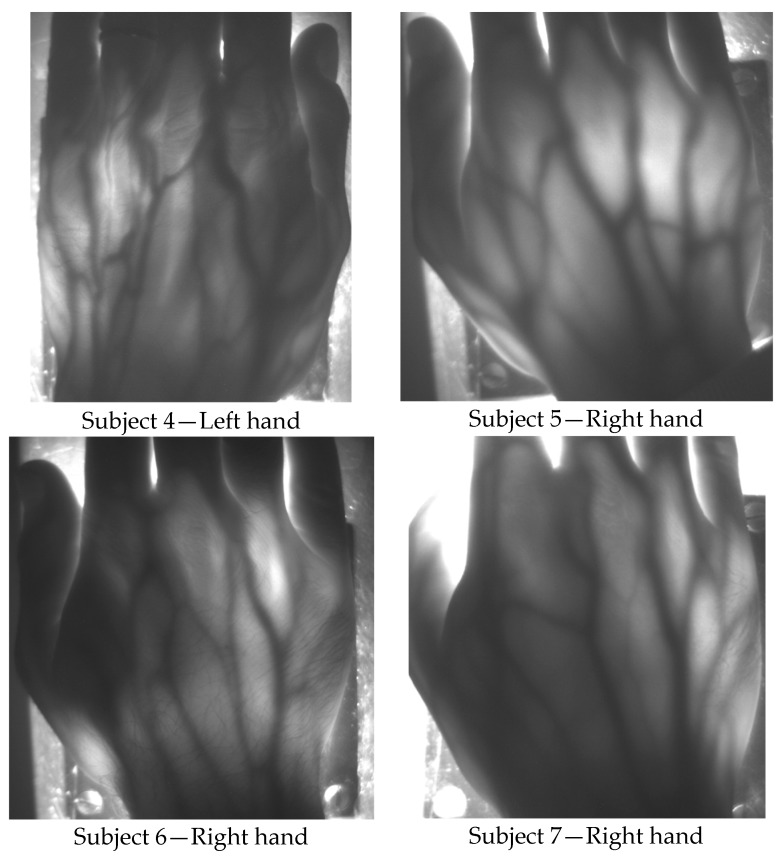
Raw pictures acquired with the transillumination system on white-skin, male subjects 4, 5, 6 and 7: morphological details are well visible in all subjects.

To demonstrate the potentiality of this system for generating low resolution images of in vivo human tissues, the instrumental configuration illustrated in Figure 1 has been employed for transilluminating the hand of human volunteers at rest and acquiring photos as well as video sequences. Before the procedure, the volunteers provided an informed consent to participate in the study and to publish their images and the detected physiological parameters. They were all able to form a reasoned decision, thanks to their scientific background and knowledge about the use of non-ionizing radiation and laser safety requirements. No serious ethical issues were raised by the ethics committee, at this stage of the research. Table 1 summarizes age, weight and height of the tested, healthy human subjects, as well as the results of the functional testing, described next. Subjects 1 to 7 have white skin, subject 8 has dark skin. During the test, the volunteers were seated and relaxed; tissues were not subjected to any kind of thermal or pressure stress. Pictures and videos were taken after manually changing the focal length of the objective lens until a sufficiently focused image appeared on the screen of the notebook. Aim of the focusing phase was to optimize the contrast between the vessels and the rest of the tissue. Pictures of the hands, taken in standard ambient day-light conditions with exposure time values ranging from 200 up to 400 ms, provided a clear visualization of the dorsal vein tree, thanks to the strong absorption of the NIR light by hemoglobin, as shown in Figure 4 and Figure 5. Figure 4 illustrates hand pictures of three white-skin, female subjects (Subjects 1, 2 and 3). Figure 4a shows a picture acquired with all VCSELs OFF, to demonstrate good rejection of ambient light. Figure 4b–d present the pictures acquired with all VCSELs ON, where morphological details are well visible. Figure 5 illustrates hand pictures of four white-skin, male subjects (Subjects 4, 5, 6 and 7). A residual granularity induced by the speckle pattern effect, though observed when capturing in reflection the light projected by the VCSEL array on white paper, cannot be appreciated in the saved photos of the transilluminated hands.

Hand veins follow the course of the corresponding arteries but arteries are located deeper in the tissue; moreover, veins are in many cases doubled compared to the corresponding artery and have transverse anastomoses, also visible in the pictures presented in Figure 4 and Figure 5. Pulsating blood flow occurs in the arteries and leads to a periodic variation of the blood volume in vessels, which in turns generates a variation of the intensity of the transmitted light [27,28]. As for PPG signals, a local increase of blood volume implies a local increase of NIR absorption: as a consequence, the gray level value of the pixels corresponding to the veins decreases and this variation can be detected by the CMOS camera. Detection of breath and hearth rate has been achieved by processing with a MatLab script the sequence of hand images obtained by recording 1 min long videos with a frame rate of 85 frames per second.

The software routine allows one to select a region of interest (ROI) where the time evolution of the spatial average of the gray level can be computed. The preferred ROI has a rectangular shape, approximately 100 × 50 pixels, with the longest side parallel to one of the main veins; it is usually selected in coincidence of a deeper artery where the pulsating blood flow occurs [27,28]. With this elaboration, we have obtained time-varying signals and we extracted their spectral content by performing the Fast Fourier Transform (FFT). Limiting our attention to the low-frequency region of the spectrum we can recognize a peak at the frequency *f*_HR_, corresponding to the expected heart rate, and another peak at a lower frequency *f*_RR_ corresponding to the expected respiratory rate. In some acquisitions, higher harmonics at 2*f*_HR_ and 3*f*_HR_ are also visible in the FFT spectrum. The heart rate in beats/min (bpm in Table 1) can be calculated by multiplying by 60 the frequency *f*_HR_ in Hz and the respiratory rate in breaths/min can be calculated by multiplying by 60 the frequency *f*_RR_. The values of heart rate in beats/min and of respiratory rate in breaths/min found in this way were found in agreement, respectively, with the heart rate attained by counting the number of heart beats, sensed manually on the wrist, and with the respiratory rate estimated by counting the breaths taken by the subject during video recording, as reported in Table 1. By processing different video sequences, taken on the same subject in similar resting conditions, spectra with similar line-shapes were attained.

**Figure 6:**
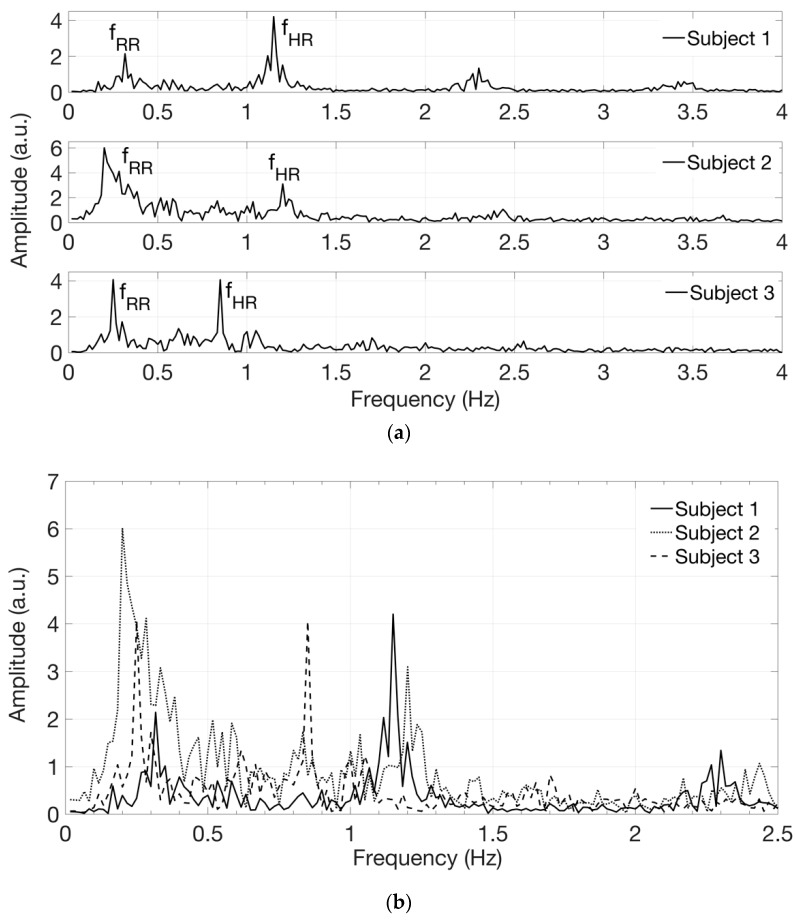
Signals in the frequency domain extracted from the acquired videos on white-skin, female subjects 1, 2 and 3. (**a**) Spectra are reported in separate graphs for each subject; (**b**) Spectra are superimposed for comparison, on a narrower frequency range.

**Figure 7:**
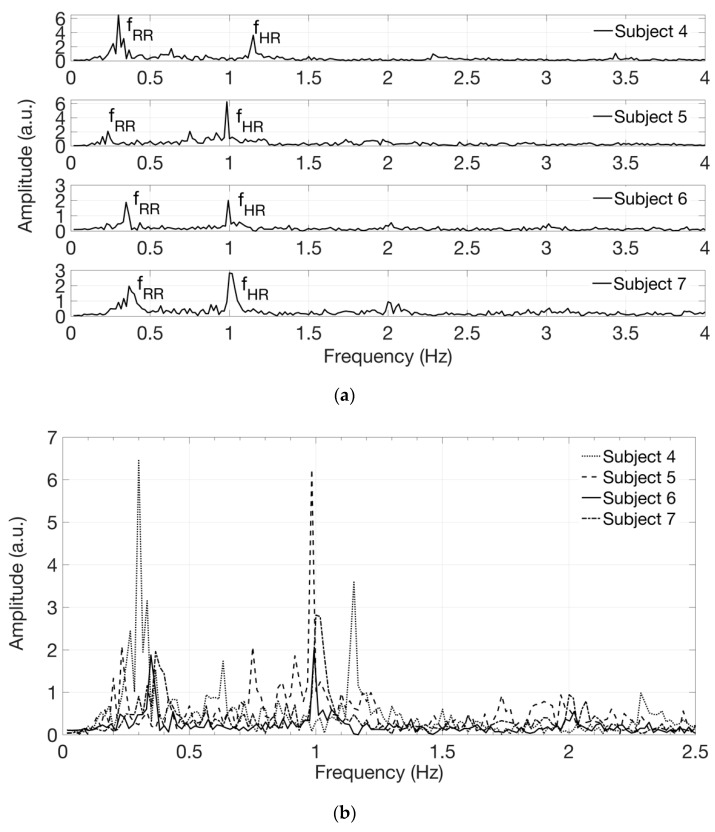
Signals in the frequency domain extracted from the acquired videos on white-skin, male subjects 4, 5, 6 and 7. (**a**) Spectra are reported in separate graphs for each subject; (**b**) Spectra are superimposed for comparison, on a narrower frequency range.

Figure 6 shows the signals in the frequency domain extracted from the acquired videos on white-skin female subjects 1 to 3. The spectra, reported in Figure 6a in separate graphs, are then superimposed for comparison in Figure 6b, displayed on a narrower frequency range, using different line styles, such as solid, dotted and dashed lines. Figure 7 shows the signals in the frequency domain extracted from the acquired videos on white-skin male subjects 4 to 7. The spectra, reported in Figure 7a in separate graphs, are superimposed for comparison in Figure 7b, displayed on a narrower frequency range, using different line styles, such as solid, dotted, dash-dotted and dashed lines. The presence of components at the frequency *f*_RR_, corresponding to the expected respiratory rate, reflects the respiratory influence on the heart rate variability, known as respiratory sinus arrhythmia [29].

**Figure 8:**
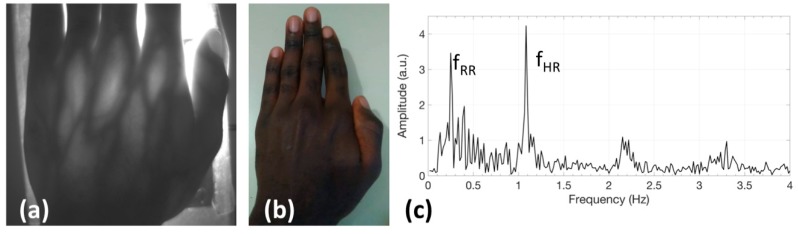
Results obtained on a dark-skin, male subject. (**a**) image of the hand acquired with the transillumination system (exposure time 306 ms); (**b**) photo of the hand taken with a color CMOS camera without NIR illumination and filter, to demonstrate the dark color of the skin of this subject; (**c**) signal in the frequency domain extracted from the video taken on this subject.

Finally, Figure 8a reports the raw image acquired with the transillumination system applied to test to a dark-skin, male subject (No. 8 in Table 1): morphological details are still visible in transillumination, although with lower contrast. A picture taken with a color CMOS camera without NIR illumination and filter is also presented in Figure 8b to demonstrate the dark color of the skin. The signal in the frequency domain, extracted from the video taken on this subject and reported in Figure 8c, clearly shows the heart and breath rate components. 

## 4. Discussion and Conclusions

We have realized a portable, optoelectronic instrumental configuration for efficient NIR transillumination and image detection of in vivo human samples, in order to obtain morphological and functional real-time images of the inner structure of diffusing non-homogeneous biological samples with thickness up to 5 cm. We have demonstrated that this system correctly detects the frequency components of heart and breath rate, without applying any pressure or other physical stress on the tissues and without inducing any photochemical reaction. Successful demonstration has been carried out on subjects with white or dark skin and normal body size, as reported in Table 1. Future work could be devoted also to investigate the limit of operation of such a system on the hand of overweight or very muscular subjects or, more in general, on thicker tissues. Thanks to the very low driving current for the VCSEL, the power dissipation is very low and the surface temperature of the illuminator increases by only a few degrees centigrade. The configuration is in principle compatible with small form factor devices, suitable for battery operation and minimally invasive for in vivo applications. It is the demonstration of a technical proof a principle paving the way for the realization and further clinical testing of diagnostic systems for non-invasive, in vivo imaging based on transillumination that can be successfully employed in a wider range of medical applications where the resolution and the penetration depth of X-rays are not required but real-time operation and non-ionizing radiation are prescribed. 

A foreseen clinical application could be in a diagnostic imaging system that substitutes X-rays for detecting pneumo-thorax in premature newborns: they are often so tiny that the total thickness of their body is of the order of a few optical penetration depths of 850 nm light. A larger matrix can be easily implemented, eventually exploiting a different spatial distribution of the sources, and a flexible support could become more appealing for medical application. The implemented configuration could be also interesting for biometric recognition and validation [30].

With regard to vital sign detection, future work will have to investigate real time visualization of parameters as well as the effects of motion artefacts, whereas an interesting application could be in the evaluation of tremor frequency characteristics in Parkinson’s disease. The results of this investigation cannot, of course, be claimed as competitors of highly sophisticated methods and complex solutions such as microscopy-based techniques or optical coherence tomography. To the best of our knowledge, this is the first demonstration of a VCSEL-based transillumination system that can acquire images and vital signs in ambient day-light without applying any physical constraint or thermal stress on the tissues.

## Figures and Tables

**Table 1:** Summary of tested subjects. * number of breaths counted in a minute; ** number of heart beats counted in a minute. Subjects 1 to 7 have white skin. Subject 8 has dark skin.

Subject No.	Sex	Age (yr)	Weight (kg)	Height (cm)	*f*_RR_ (Hz)	*f*_RR_ × 60 (bpm)	Counted Breaths *	*f*_HR_ (Hz)	*f*_HR_ × 60 (bpm)	Counted Heart Beats **
1	F	56	59	165	0.317	19.00	19	1.150	71.04	71
2	F	24	53	163	0.200	12.00	12	1.201	72.06	71
3	F	22	58	165	0.250	15.00	15	0.850	51.00	51
4	M	63	76	187	0.300	18.00	18	1.151	69.06	69
5	M	19	64	186	0.233	14.00	14	0.984	59.04	59
6	M	42	74	176	0.348	20.88	21	0.993	59.58	60
7	M	41	75	178	0.367	22.02	22	1.000	60.00	60
8	M	22	76	175	0.250	15.00	15	1.084	65.04	65

## References

[1] Fu X., Dhawan A.P., D’Alessandro B. (2010). Optical Imaging Modalities for Biomedical Applications. IEEE Rev. Biomed. Eng..

[2] Boas D.A., Pitris C., Ramanujam N. (2011). Handbook of Biomedical Optics.

[3] Bigio I.J., Fantini S. (2016). Quantitative Biomedical Optics: Theory, Methods and Applications.

[4] Jacques S.L. (2009). Spectral imaging and analysis to yield tissue optical properties. J. Innov. Opt. Health Sci..

[5] Jacques S.L. (2013). Optical properties of biological tissues: A review. Phys. Med. Biol..

[6] Anderson R.R., Parrish J.A. (1981). The optics of human skin. J. Invest. Dermatol..

[7] Drexel W., Fujimoto J.G. (2015). Optical Coherence Tomography: Technology and Applications.

[8] Chacko S., Singh M. (2000). Three-dimensional reconstruction of transillumination tomographic images of human breast phantoms by red and infrared lasers. IEEE Trans. Biomed. Eng..

[9] Diakides M., Bronzino J.D., Peterson D.R. (2017). Medical Infrared Imaging: Principles and Practices.

[10] Andersson-Engels S., Berg R., Svanberg S., Jarlman O. (1990). Time-resolved transillumination for medical diagnostics. Opt. Lett..

[11] Berg R., Jarlman O., Svanberg S., Jarlman O. (1993). Medical transillumination imaging using short-pulse diode lasers. Appl. Opt..

[12] Srinivasan R., Singh M. (2003). Laser backscattering and transillumination imaging of human tissues and their equivalent phantoms. IEEE Trans. Biomed. Eng..

[13] Durduran T., Choe R., Baker W.B., Yodh A.G. (2010). Diffuse optics for tissue monitoring and tomography. Rep. Prog. Phys..

[14] Gonzalez J., Roman M., Hall M., Godavarty A. (2012). Gen-2 Hand-Held Optical Imager towards Cancer Imaging: Reflectance and Transillumination Phantom Studies. Sensors.

[15] Jung Y.J., Roman M., Carrasquilla J., Erickson S.J., Godavarty A. (2015). Non-contact deep tissue imaging using a hand-held near infrared optical scanner. J. Med. Diagn. Meth..

[16] Kim D., Kim Y., Yoon S., Lee D. (2017). Preliminary Study for Designing a Novel Vein-Visualizing Device. Sensors.

[17] ASTODIA^®^ DIAPHANOSCOPE. http://www.stihlerelectronic.de/products/diaphanoscopy.html.

[18] Breastlight^TM^ Home Page. http://www.breastlight.com/.

[19] Moraes J.L., Rocha M.X., Vasconcelos G.G., Filho J.E.V., De Albuquerque V.H.C., Alexandria A.R. (2018). Advances in Photopletysmography Signal Analysis for Biomedical Applications. Sensors.

[20] Kumar M., Veeraraghavan A., Sabharwal A. (2015). DistancePPG: Robust non-contact vital signs monitoring using a camera. Biomed. Opt. Express.

[21] Lazović B., Mazic S., Zikich D., Žikić D. (2015). The mathematical model of the radial artery blood pressure waveform through monitoring of the age-related changes. Wave Motion.

[22] Rebohle L., Gebel T., Yankov R., Trautmann T., Skorupa W., Sun J., Gauglitz G., Frank R. (2005). Microarrays of silicon-based light emitters for novel biosensor and lab-on-a-chip applications. Opt. Mater..

[23] Xu K. (2016). Integrated Silicon Directly Modulated Light Source Using P-Well in Standard CMOS Technology. IEEE Sens. J..

[24] Xu K. (2018). Monolithically integrated Si gate-controlled light-emitting device: Science and properties. J. Opt..

[25] Germer S., Cherkouk C., Rebohle L., Helm M., Skorupa W. (2013). Si-based light emitter in an integrated photonic circuit for smart biosensor applications. SPIE Microtechnologies.

[26] TTElectronics. http://www.ttelectronics.com/sites/default/files/download-files/Datasheet_OPV332.pdf.

[27] Kopf-Maier P. (2000). Anatomia umana. Atlante di Wolf-Heidegger: 1.

[28] Tazzi A., Montagnani S. (2006). Trattato di Anatomia Umana, Volume 1.

[29] Hirsch J.A., Bishop B. (1981). Respiratory sinus arrhythmia in humans: how breathing pattern modulates heart rate. Am. J. Physiol.-Heart Circ. Physiol..

[30] Crisan S., Jiang R., Al-maadeed S., Bouridane A., Crookes P., Beghdadi A. (2017). A Novel Perspective on Hand Vein Patterns for Biometric Recognition: Problems, Challenges, and Implementations. Biometric Security and Privacy, Signal Processing for Security Technologies.

